# Hybrid Zeolite SAPO-34 Fibres Made by Electrospinning

**DOI:** 10.3390/ma11122555

**Published:** 2018-12-15

**Authors:** Angela Malara, Patrizia Frontera, Lucio Bonaccorsi, Pier Luigi Antonucci

**Affiliations:** 1 Department of Civil, Energetic, Environmental and Materials Engineering, Mediterranean University of Reggio Calabria, Reggio Calabria, 89134 Italy; patrizia.frontera@unirc.it (P.F.); lucio.bonaccorsi@unirc.it (L.B.); pierluigi.antonucci@unirc.it (P.L.A.); 2 Consorzio Interuniversitario per la Scienza e la Tecnologia dei Materiali (INSTM), 50121 Firenze, Italy; 3 CNR-ICCOM Institute of Chemistry of Organometallic Compounds, 56124 Pisa, Italy

**Keywords:** microfibres, electrospinning, adsorption, SAPO-34, solar cooling

## Abstract

A new generation of compressor-free heat pumps based on adsorption technology and driven by solar energy is available. Performance and costs are, however, the main obstacles to their commercial diffusion, and more material and system developments are required. In this work, a new coating made of microfibres produced by the electrospinning of polymer/zeolite mixtures is presented. Three different polymer carriers, polyvinyl acetate, polyethylene oxide and polystyrene, have been used together with zeolite SAPO-34 as an adsorbing material. Electrospun microfibres showed a mean diameter ranging from 0.75 μm to 2.16 μm depending on the polymer carrier, with a zeolite content from 60 wt.% to 87 wt.%. Thermal analysis (TGA-DSC) results showed that water desorption from microfibres at T = 150 °C was close to 17 wt.%, a value in agreement with the adsorption capacity of pure SAPO-34. The morphology characterization of coatings demonstrated that the microfibre layers are highly porous and have an elevated surface area.

## 1. Introduction

Zeolites are a class of nanoporous inorganic materials that are widely used in several industrial processes, such as molecular sieves, catalysts, adsorbents and ionic-exchangers. Since the early 1970s, zeolites have been the preferred porous material for adsorption heat pumps and chillers [1,2,3] instead of other adsorbent materials [4,5]. In these systems, the mechanical compression of the refrigerating fluid that drives the reversed Carnot cycle is replaced by the adsorption of water vapour on a bed of zeolites, which is regenerated by waste heat recovered from thermal energy sources. Detailed descriptions of adsorption heat pumps and their thermodynamic functioning are reported in the literature [6,7,8]. In the last few years, a new interest in this application grew due to the attractive possibility of coupling an adsorption heat pump to solar cells in order to produce air-conditioning for homes and offices by solar energy [9,10,11]. The commercial development of heat pumps and chillers is, however, related to the availability of materials and systems that can compete in terms of performance and cost with the traditional, electrically-driven heat pumps. Several efforts have been made to improve thermodynamic efficiency by decreasing thermal and mass transfer resistances in the adsorbing component. One fundamental topic is the configuration of the porous material inside the heat pump apparatus. In earlier systems, the zeolite was used in the form of millimetric-sized pellets to fill the free spaces between the fins of the heat exchanger used to transfer/receive thermal energy to/from the adsorbing material. In this configuration, the poor contact at the pellets’ metal interface caused elevated heat transfer resistance, thus lowering the general efficiency of the heat pump [2,3,12]. Better solutions have been proposed and studied, including the direct synthesis of zeolite layers on the heat exchanger surface and the development of binder-base coatings [13,14,15], composite coatings or hybrid foams [16,17], each with its own advantages and disadvantages. Through direct synthesis, the zeolite crystals are grown on the metal surface and the interface contact is ideal. However, the chemical reaction imposes limits on the coating thickness attainable due to the coprecipitation of unwanted phases. Bonded and composite coatings are easier to obtain but are generally dense. Thus, the resistance to vapour diffusion is high, while foamed coatings have a high porosity but are more difficult to produce.

In this work, we propose a new coating material made of hybrid zeolite/polymer microfibres produced by electrospinning. The microfibre coating shows important advantages compared to the discussed solutions, due to the peculiarity of the layers formed by electrospinning. The intrinsic high surface area per weight of the microfibres’ coating assures an improved interface contact between zeolite granules and the metal surface compared to the use of zeolite pellets or compact coatings, while the elevated porosity of the electrospun layers allows the increased coating thickness maintaining low pressure to drop to the vapour flow.

Electrospinning is a technique to produce nano- and microfibres, which uses an electric force to draw charged threads of polymer solutions up to fiber diameters on a nanometric scale. With the help of a suitable polymeric vector, it is possible to produce nanofibres of organic, inorganic and hybrid composite materials. This is a very simple, scalable and inexpensive technique used to produce one-dimensional (1D) nanostructures, and allows us to obtain different morphologies/architectures, such as dense, hollow, core-shell and assemblies’ fibres [18]. Briefly, the electrospinning technique is based on the application of an electric field to a drop of the polymer carrier solution on the tip of a spinneret. Increasing the intensity of the electric field causes the drop to elongate and form a conical shape (the Taylor cone) [18]. When the applied electric field reaches a critical value, the repulsive electric forces overcome the surface tension of the drop. Then, a charged jet of the solution is expelled from the tip of the cone and accelerates downfield. Between the tip and the collector, there is an electromechanical instability of the jet, which leads to a further elongation of the liquid filament and the evapouration of the solvent to generate solid micro- or nanofibres on the grounded collector [18]. The field of applications of electrospun nanostructures is large, covering water treatment to biomedical, sensing, catalyst and electronic applications [19,20,21,22,23,24,25,26]. Recently, this technique has been intensively employed for the design and fabrication of structured nanofibrous materials for energy conversion and storage devices, as well as dye-sensitized solar cells, fuel cells, lithium/sodium ion batteries and supercapacitors [27].

In this study, the microfibres were obtained from mixtures of zeolite SAPO-34 with three different polymer carriers. This zeolite was the most promising nanoporous material for adsorption applications driven by solar sources [28]. Results demonstrated that hybrid microfibres maintained the adsorption properties to water vapour of SAPO-34, were thermally stable and had a morphology particularly apt to create adsorptive coatings on the heat exchanger surface.

## 2. Experimental

Zeolite fibres have been obtained by electrospinning of hybrid solutions of SAPO-34 and three different polymers: Polyvinyl acetate (PVA), polyethylene oxide (PEO) and polystyrene (PS), according to the following procedures.

### 2.1. Zeolite SAPO-34 Synthesis

SAPO-34 powder was prepared by hydrothermal synthesis of an aqueous solution of composition: 1Al_2_O_3_:1P_2_O_5_:0.6SiO_2_:0.7TEA_2_O:70H_2_O, maintained at T = 200 °C for 72 h in a stainless steel autoclave. Details of the synthesis procedure are reported elsewhere [11]. After synthesis, the solution was centrifuged and washed with distilled water several times to separate the precipitated powder. Finally, the SAPO zeolite was heated in the oven at T = 550 °C for 6 h to eliminate the organic template. The zeolite powder was characterized by X-ray diffraction (XRD, Bruker D2 Phaser, Karlsruhe, Germany) and scanning electron microscopy (SEM Phenom ProX, Deben, Suffolk, UK).

### 2.2. Electrospinning of Hybrid Microfibres

The production of SAPO-34 fibres by electrospinning consisted of preparation of a precursor solution made by mixing the polymeric component, the solvent and the zeolite powder. Polyethylene oxide (PEO, M_w_ = 600,000, Sigma Aldrich, Saint Louis, Missouri, Stati Uniti) and polyvinyl acetate (PVA, M_w_ = 500,000, Sigma Aldrich) polymers were dissolved in ethanol (EtOH), 8% and 12% *w*/*w*, respectively, whereas dimethylformamide (DMF) acted as solvent for polystyrene (PS, M_w_ = 192,000, Sigma Aldrich), 15% *w*/*w*. Polymeric solutions were stirred at room temperature for 3 h. Finally, zeolite powder was added according to the weight % reported in Table 1. Each mixture was further stirred for 2 h, then loaded into a 5 cc syringe fitted with a 0.7 mm steel needle and driven by a syringe pump at a flow rate of 1.5 mL/h. The applied voltage was 13 kV and the distance between the needle tip and the collector was 12 cm. These process parameter values have been chosen after several experiments devoted to the optimization of the nanofibres morphology.

Table 1 contains the formulations adopted with the different polymers as well as different SAPO-34 concentrations tested.

The data in Table 1 shows four mixtures with different zeolite contents, from 60 to 87 wt.%, prepared for each polymeric component. However, not all the polymer/zeolite combinations resulted in an electrospinnable mixture. Using polystyrene (PS), for instance, when the zeolite content was > 60 wt.%, the obtained microfibres were highly irregular with an abundant formation of lumps of polymer and zeolite. Similarly, for other combinations (n/a in Table 1), the results were not considered because the electrospun material was unacceptable in terms of morphology. The objective required by the application was to maximize the zeolite weight in the microfibres coating. Therefore, for the sake of conciseness, only the satisfactory results will be discussed below.

### 2.3. Zeolite Microfibres Characterization

The synthesized SAPO-34 was analysed by XRD in the 2θ range 5–40° (Cu K_α1_ = 1.54056 Å) to verify the zeolite crystallinity and the presence of amorphous or extraneous phases. SEM was used to characterize the zeolitic powder and the electrospun fibres’ morphology. The microfibre dimensions were obtained by image analysis using the software ImageJ [29] and the plugin DiameterJ [30], specifically developed for nanofibre characterization.

TGA/DSC analysis (STA 409 PC Netzsch, Selb, Germany) was carried out on the pure zeolite and the hybrid microfibres by increasing temperature from 25 °C to 500 °C at a rate of 10 °C/min, under a nitrogen atmosphere.

## 3. Results and Discussion

**Figure 1:**
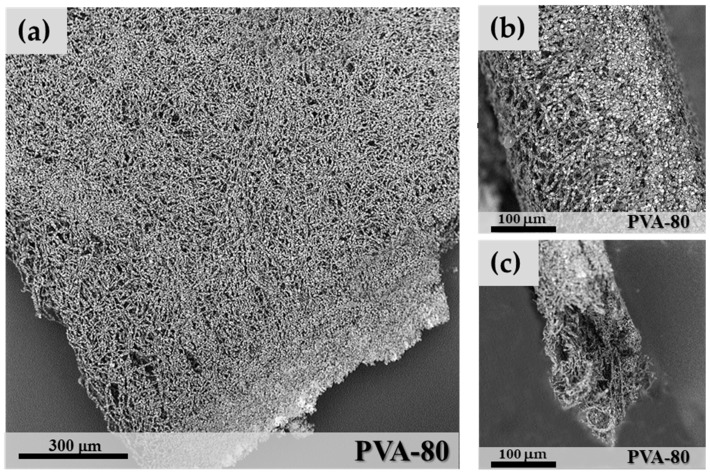
SEM image of a microfibre coating of sample PVA-80 (**a**); Coating cross-section (**b**) and a fracture surface (**c**).

In Figure 1, a coating made of microfibres obtained by electrospinning is shown. The microfibres that form the coating appear to be closely interconnected, although a large porosity of the layer is maintained, which allows a high-vapour permeability. The zeolite is regularly distributed as individual granules along all the microfibres with a significant increase of the surface area available to vapour adsorption. Four grams of SAPO-34 correspond to about 30 pellets of 5 mm diameter. However, when electrospun as microfibres, a hybrid microfibre mat of side = 190 mm× 190 mm and thickness = 0.5 mm is created. Other coating techniques, such as binders or direct synthesis, always result in more compact and denser layers. An improvement of the mechanical properties of the microfibres’ coating is also expected. Zeolite coatings are generally brittle due to the presence of the zeolite crystals, which act as defects in the layer. The fibrous nature of the fracture surface produced in the mat (Figure 1c) suggests a nonbrittle progression of the fracture.

### 3.1. Adsorbing Material Characterization

**Figure 2:**
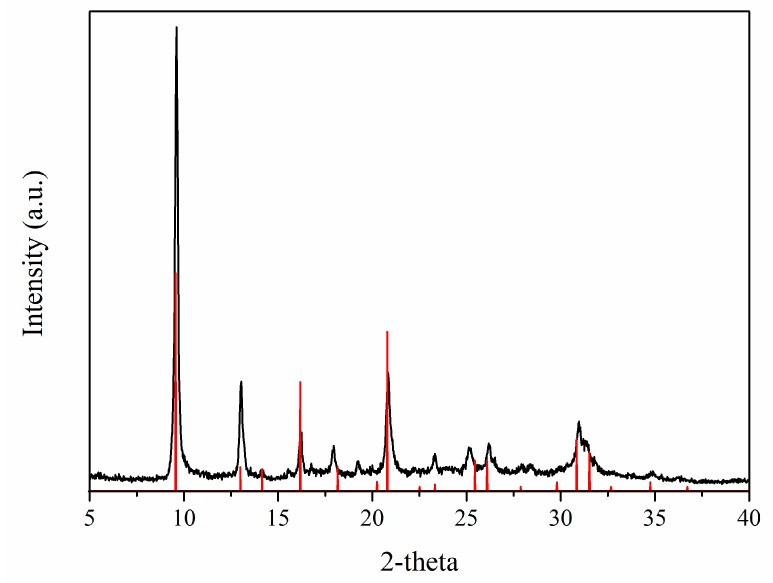
X-ray diffractogram of the synthesized SAPO-34. Reference 2-theta values are shown for comparison [32].

**Figure 3:**
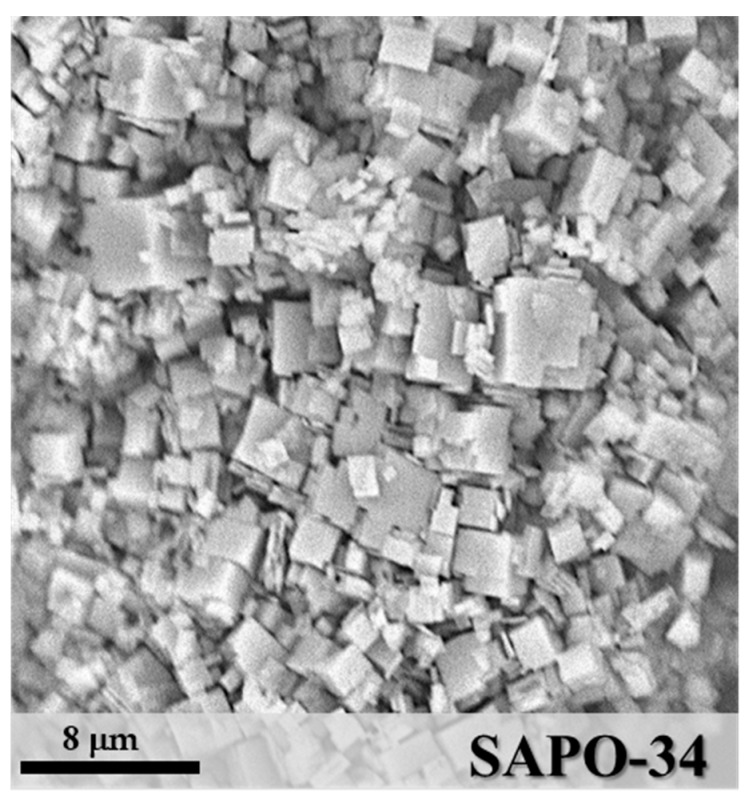
SEM image of the synthesized SAPO-34.

SAPO-34 is a microporous crystalline material with a chabazite framework formed by Al–O–P tetrahedra, where some P positions are substituted by Si atoms [29,31]. The X-ray diffractogram (Figure 2) shows the pattern of the synthesized powder after activation, confirming the formation of pure SAPO-34 crystals with the typical cubic morphology and a particle size in the range of 1–4 μm, as shown by the SEM image (Figure 3).

**Figure 4:**
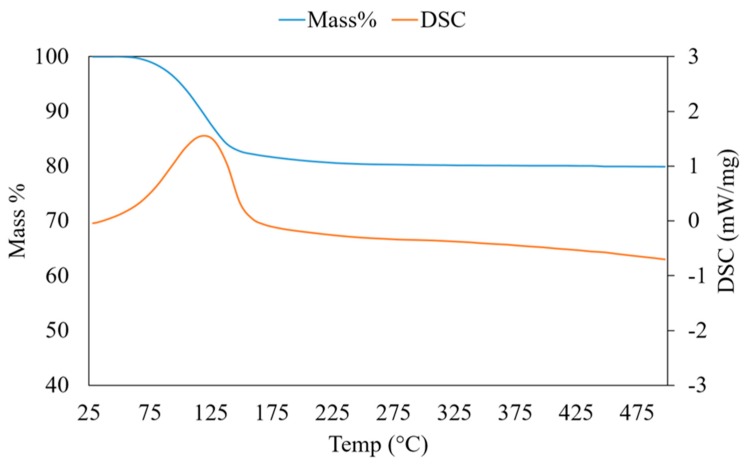
Thermal analysis (TGA-DSC) of the synthesized SAPO-34.

The framework of SAPO-34 showed a negative charge consequent to the substitution of P, which gives this zeolite the ability of adsorbing water vapour up to 30% by weight [11,31]. The thermo-gravimetric curve of the synthesized SAPO-34 (Figure 4) shows that the water desorption begins at T ~ 50 °C and concludes at a relatively low temperature (250 °C). As shown in Figure 4, the maximum weight decrease observed at atmospheric pressure is 17.5 wt.%, while complete water desorption is possible only in a high vacuum [31]. The calorimetric curve in Figure 4 shows one peak at 110 °C, due to the endothermic desorption of water. For the remaining temperature increase, no evidence of material transformation is observed. The most important feature of SAPO-34 that makes this zeolite the preferred material for adsorption heat pumps is the narrow range of desorption temperatures, between 50 and 150 °C, where most of the water molecules trapped in the pores is released [33].

### 3.2. Polyethylene Oxide/SAPO-34 Microfibres

**Figure 5:**
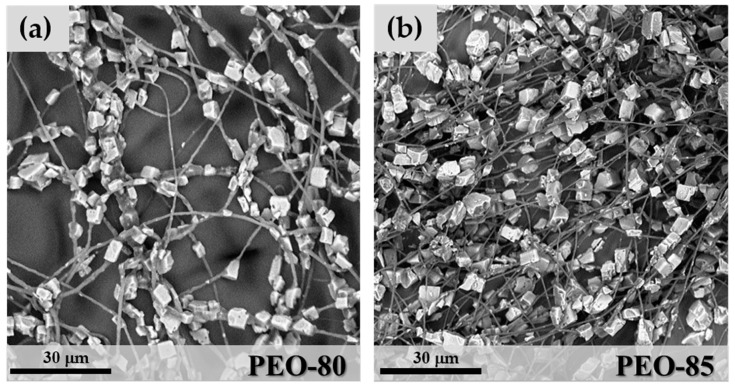
SEM images of sample PEO-80 (**a**) and PEO-85 (**b**).

At the lowest zeolite content used, corresponding to 60 wt.% of SAPO-34 and 40 wt.% of PEO solution, the hybrid microfibres showed a significant segregation of the inorganic crystals, which resulted in an irregular distribution in a matrix of polymeric microfibres (not shown). By increasing the SAPO-34 percentage, a better mixing of the polymeric phase and zeolite crystals was obtained. The hybrid fibre morphology was characterized by a regular distribution of zeolite crystals along the polymeric fibres (Figure 5). At 80 wt.% zeolite concentration, however, some irregular microfibres were observed (Figure 5a), due to the formation of thick aggregates of crystals in which the fibrous structure was mostly lost. The mean fibre diameter for sample PEO-80 was 1.06 μm (StdDev = 0.44) and was measured by image analysis on cross-sections of fibre segments free of crystals. By increasing SAPO-34 content to 85 wt.% (sample PEO-85 in Figure 5b), the particle distribution improved and more regular hybrid microfibres were observed. The microfibres show a “necklace” morphology, where the zeolite crystals were regularly distributed along filaments of the polymeric matrix (Figure 5b). The mean fibre diameter for PEO-85 samples was 0.75 μm (StdDev = 0.38), slightly lower than in PEO-80.

**Figure 6:**
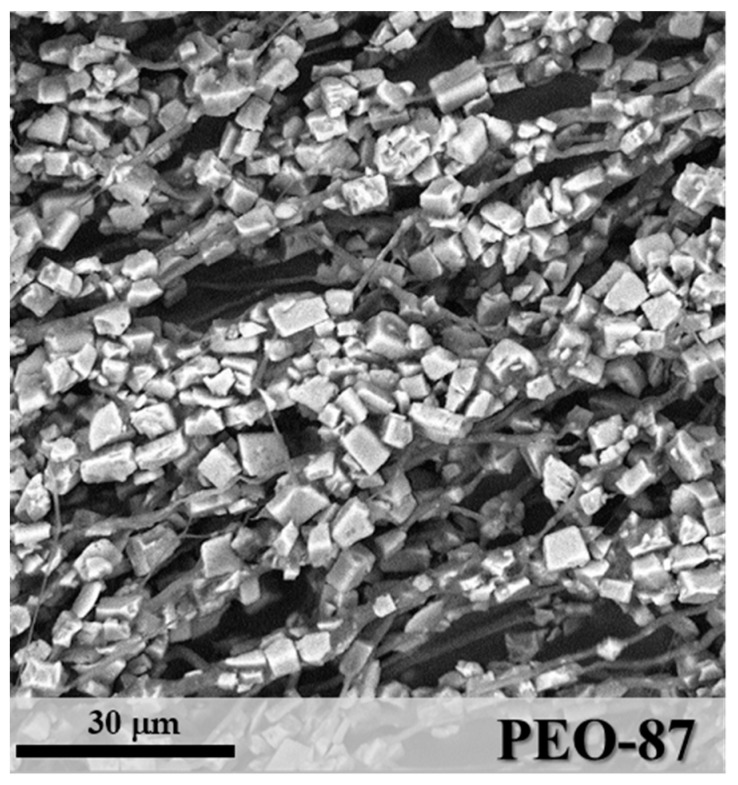
SEM image of PEO-87.

When the zeolite concentration reached 87 w% (sample PEO-87 in Figure 6), the layer morphology was determined by a significant increase of aggregates of SAPO-34 particles through the microfibres. In this case, the mean diameter of fibre segments was 2.16 μm (StdDev = 1.21), a value influenced by the irregular particle distribution. By increasing the zeolite content in the mixture, a more difficult homogenization between inorganic and polymeric components was observed.

### 3.3. Polyvinyl Acetate/SAPO-34 Microfibres

**Figure 7:**
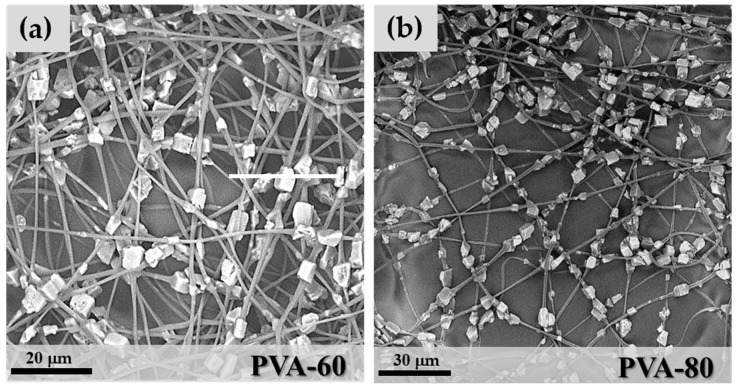

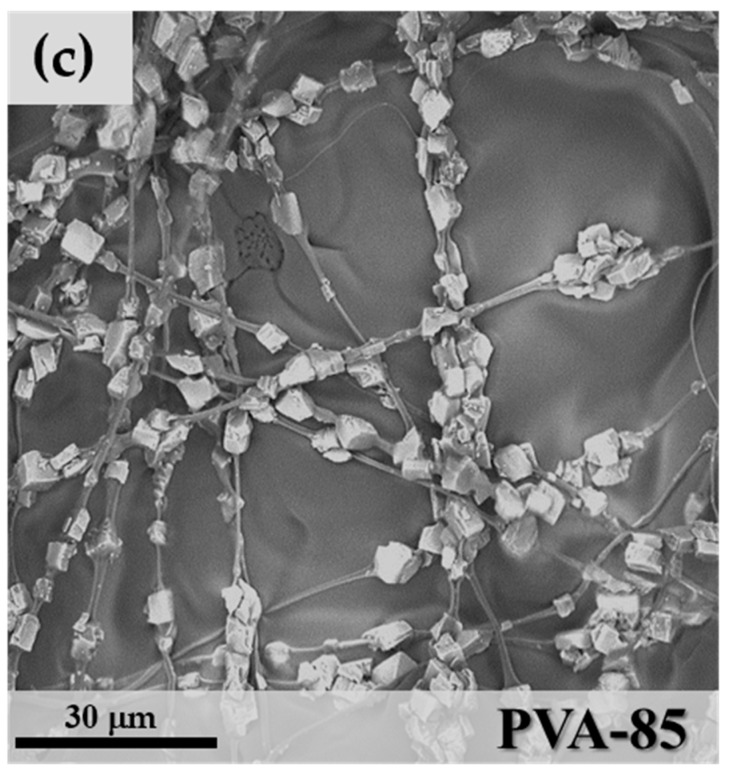
SEM images of (**a**) PVA-60; (**b**) PVA-80 and (**c**) PVA-85.

PVA mixtures were spinnable from 60 wt.% to 85 wt.% zeolite contents, but not at the highest value of 87 wt.%. In the last case, an excessive increase of the solution viscosity was the main reason for the formation of particle aggregates and the loss of the fibrous nature of the spun layers. PVA 60, 80 and 85 wt.% microfibres maintained the necklace morphology of PEO-85, as shown in Figure 7.

**Figure 8:**
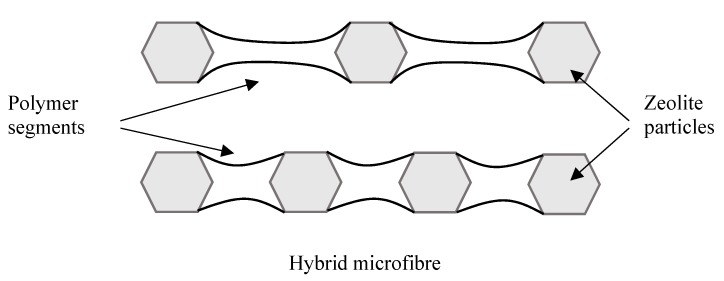
Scheme of a microfibre as the number of zeolite crystals increases.

Particle clumping was less evident with polyvinyl acetate due to improved wettability between the polymer matrix and zeolite granules, and the formation of mixtures with higher stability during the electrospinning process. One important characteristic of the precursor mixture is the capability to maintain a homogeneous distribution of the zeolite crystals in the polymeric solution throughout the whole electrospinning process. The aggregation of crystals with the consequent formation of thick and irregular fibres was observed only at the concentration of 85 wt.% (Figure 7c). Comparing the mean fibre diameter of the three samples: D_PVA-60_ = 0.97 μm (StdDev = 0.37), D_PVA-80_ = 1.18 μm (StdDev = 0.45) and D_PVA-85_ = 1.27 μm (StdDev = 0.78), an increase in the cross-section of microfibres with zeolite concentration was detected. This can be considered an indication of the increase in the number of particles per fibre because the particles-free fibre segments became shorter and consequently thicker due to surface tension effects, as shown in the scheme in Figure 8.

### 3.4. Polystyrene/SAPO-34 Microfibres

**Figure 9:**
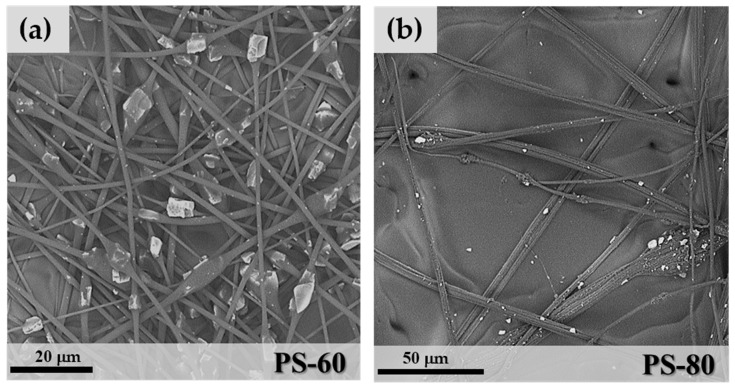
SEM images of PS-60 (**a**) and PS-80 (**b**).

As shown in Table 1, PS hybrid fibres were more difficult to produce. The only acceptable microfibres were obtained from sample PS-60 (Figure 9). Increasing the zeolite weight percentage in the polymeric solution caused a significant increase of the solution viscosity, with a consequent formation of irregular, large and aggregates of SAPO-34 granules embedded in the PS matrix (Figure 9b). PS-60 fibres in Figure 9a show a more regular morphology. However, the necklace structure of PEO and PVA samples turned into a “beaded” fibre structure [34], where the zeolite crystals are included in beads of PS, rather than regularly distributed along the fibres. In this case, the mean fibre diameter of free segments was 1.17 μm (StdDev = 0.47), a value similar to the PVA-80 sample.

### 3.5. Thermogravimetric Characterization of Hybrid Microfibres

**Figure 10:**
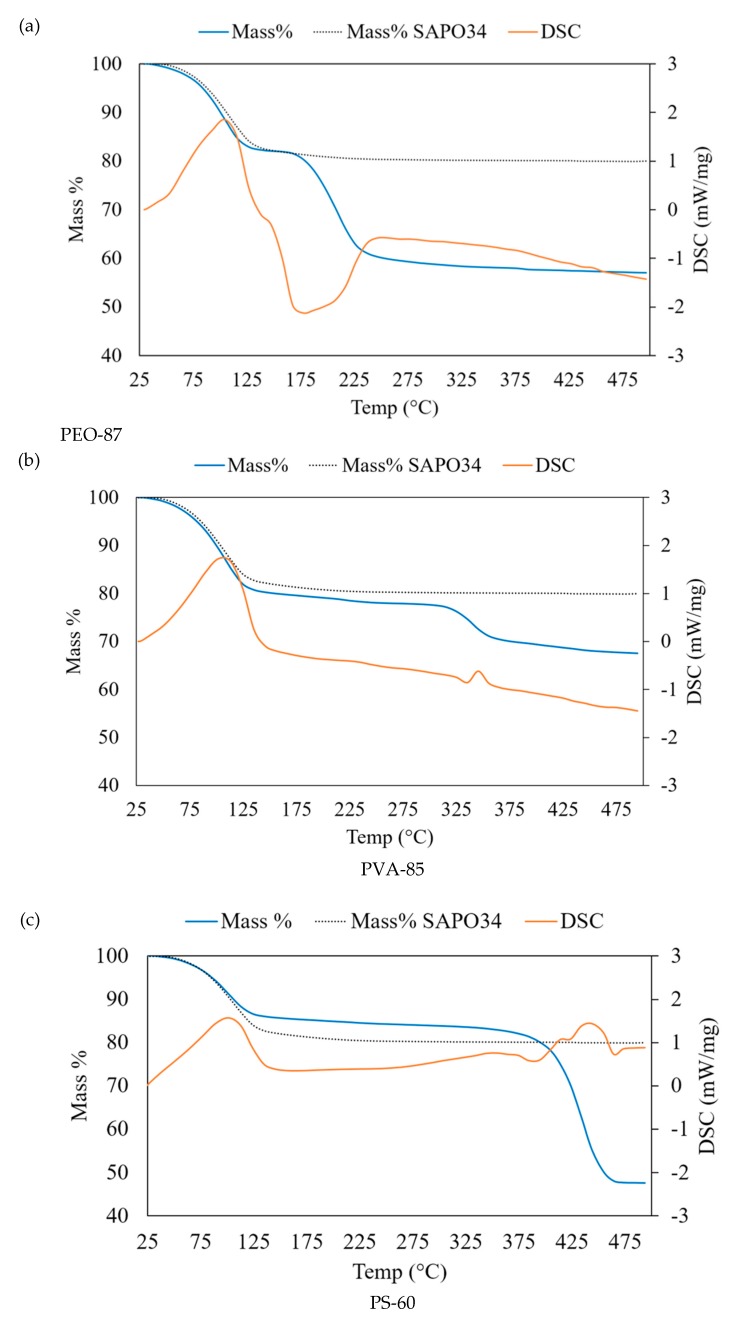
TGA-DSC curves of (**a**) PEO-87, (**b**) PVA-85 and (**c**) PS-60. TGA of SAPO-34 is shown for comparison.

ThermoGravimetric Analysis – Differential Scanning Calorimetry TGA-DSC analysis of microfibres was used to highlight any effects of pore closing of the zeolite crystals due to the polymer matrix. The main objective was to maintain the adsorption/desorption properties of SAPO-34. Another important characteristic evaluated by TGA-DSC is the thermal stability of the microfibre coatings in the operating conditions of an adsorption cycle and the degradation with temperature of the polymeric phase. For solar cooling applications, the adsorbing material is cyclically heated and cooled between 150 and 30 °C. For a complete characterization, the final temperature in TGA experiments was 500 °C. In Figure 10, the thermogravimetric curves of hybrid microfibre samples of the three polymers at their highest zeolite content, i.e., PEO-87, PVA-85 and PS-60 samples, are shown. For comparison, each plot reports the TGA curve of the pure SAPO-34 (shown in Figure 4). In the thermogravimetric curves of Figure 10, the mass decreases up to 150 °C, corresponding to the endothermic peak in DSC curves. This has to be attributed to the water desorption from the zeolite pores, in agreement with the TGA-DSC curve of SAPO-34 shown in Figure 4. The comparison demonstrates that the SAPO-34 porosity was not occluded by the polymeric component due to the particular morphology obtained in the microfibres where the zeolite crystals were regularly distributed.

The thermal degradation of the polymeric components started at higher temperatures for all the three samples presented in Figure 10. PEO is the polymer that showed the lower thermal stability. At the limit temperature T ~ 175 °C, an evident exothermic peak corresponding to the polymer oxidation was observed in the DSC curve (Figure 10a). PVA, instead, started to degrade at T > 275 °C and was completely oxidized in the temperature range of 325–375 °C, as highlighted by the exothermic peak of the DSC curve (Figure 10b). The more thermally resistant polymer was PS, showing an evident degradation at T > 375 °C in correspondence to the exothermic reaction observed in DSC.

For a more complete analysis of TGA-DSC data, the residual masses at T = 500 °C were compared with the initial compositions in Table 2. At T = 500 °C, all of the polymeric component was decomposed and the mass % attributed to the dehydrated zeolite. In Table 2, the values of the expected zeolite concentration on a dry basis and the recalculated (hydrated) zeolite % in the microfibre are reported. In PEO-87, for example, there is a difference between the added quantity of SAPO-34 (87%) and the zeolite that was effectively found in the electrospun microfibres (70.1%), which corresponds to a lack of zeolite of 19.4%. This difference is due to a partial precipitation of the zeolite particles from the solution during the electrospinning process. Data in Table 2 demonstrated that the PVA-85 final composition was the closest to the initial composition with a difference of 3.7%, confirming that polyvinyl acetate is the polymer with the highest chemical affinity with SAPO-34 particles.

## 4. Conclusions

In this study, a coating of hybrid microfibres for water adsorption applications made of SAPO-34/polymer composite and produced by electrospinning were presented and characterized. SAPO-34 was purposely synthesized and mixed with solutions of PEO, PVA and PS at different concentrations in order to obtain microfibres with high zeolite content, good adsorption properties and thermal stability. Results demonstrated that, due to the particular morphology of the electrospun microfibres, the zeolite porosity was always preserved. However, polyvinyl acetate showed higher chemical affinity to SAPO-34 zeolite, resulting in more electrospinnable solutions, better morphology and higher yield in zeolite % in the final product.

## Figures and Tables

**Table 1:** Precursor mixtures compositions.

	Zeolite/Polymeric Solution (Weight %)				Polymer	Solvent
	60%	80%	85%	87%		
Samples	n/a	PEO-80	PEO-85	PEO-87	PEO	EtOH
	PVA-60	PVA-80	PVA-85	n/a	PVA	EtOH
	PS-60	n/a	n/a	n/a	PS	DMF

**Table 2:** Calculated zeolite composition of microfibres from residual mass at 500 °C.

Microfibres SAMPLE	Initial Zeolite %	Residual Mass at T = 500 °C (Dry Weight %)		Recalculated Zeolite %	Weight Loss %
		Measured	Expected		
PEO-87	87	57.1	71.8	70.1	19.4
PVA-85	85	67.5	70.1	81.8	3.7
PS-60	60	47.5	49.5	57.6	4
